# Toward Foundation Models for Mobility Enriched Geospatially Embedded Objects

**DOI:** 10.1145/3748636.3760459

**Published:** 2025-12-12

**Authors:** Maria Despoina Siampou, Shang-Ling Hsu, Shushman Choudhury, Neha Arora, Cyrus Shahabi

**Affiliations:** † University of Southern California, Los Angeles, California, USA; ‡ Google Research, Mountain View, California, USA

**Keywords:** foundation models, GeoAI, representation learning, spatio-temporal modeling, human mobility, spatio-temporal reasoning

## Abstract

Recent advances in large foundation models (FMs) have enabled learning general-purpose representations in natural language, vision, and audio. Yet geospatial artificial intelligence (GeoAI) still lacks widely adopted foundation models that generalize across tasks that require joint reasoning over geospatial objects and human mobility. Such tasks are crucial as mobility, along with satellite imagery, street view, and text, is a core modality for understanding the physical world. We argue that a key bottleneck is the absence of unified, general-purpose, and transferable representations for geospatially embedded objects (GEOs). Such objects include points, polylines, and polygons in geographic space, enriched with semantic context and critical for geospatial reasoning. Much current GeoAI research compares GEOs to tokens in language models, where patterns of human movement and spatiotemporal interactions yield contextual meaning similar to patterns of words in text. However, modeling GEOs introduces challenges fundamentally different from language, including spatial continuity, variable scale and resolution, temporal dynamics, and data sparsity. Moreover, privacy constraints and global variation in mobility further complicates modeling and generalization. This paper formalizes these challenges, identifies key representational gaps, and outlines research directions for building foundation models that learn behavior-informed, transferable representations of GEOs from large-scale human mobility data, as well as static contextual information such as points of interest, object shapes and spatio-temporal semantics.

## 1 Introduction

Large foundation models have transformed natural language processing and computer vision by enabling models to learn contextual, general-purpose representations of words and images. These models, trained on vast amounts of publicly available data, can capture complex semantic and structural relationships and solve a wide range of downstream tasks with minimal task-specific supervision. The core idea behind these successes is that of a single model serving as a flexible backbone for many applications by leveraging transferable representations [6].

Despite these advances in other domains, GeoAI has yet to see comparable progress [12, 15, 24, 31, 46]. We argue that a key challenge lies in learning representations for geospatial objects that capture their differences in geometry, from points (e.g., business locations) to polylines (e.g., street segments) to polygons (e.g., building footprints), as well as their semantic attributes (e.g., building function). These objects are essential for globally effective geospatial reasoning tasks, such as determining whether a coffee shop is located within a mall or computing the distance from a point of interest (POI) to the nearest road. We introduce the term **Geospatially Embedded Objects (GEOs)** to refer to these entities: points, polylines and polygons situated in geographic space, enriched with semantic context, that are integral to spatial reasoning.

The analogy to language is intuitive: just as words derive meaning from their context within sentences, GEOs can gain contextual meaning from patterns of human movement over time, with sequences of interactions with GEOs resembling sentences [9]. This analogy has motivated efforts to apply LLMs to learn GEO embeddings from trajectories [32, 33]. However, the analogy breaks down in practice: language-based techniques struggle to capture the unique characteristics of GEOs. *Unlike words, GEOs are embedded in continuous space, vary in scale, exhibit complex temporal dynamics, and are visited sparsely and unevenly.* Additionally, modeling GEOs introduces distinct challenges, such as privacy concerns in mobility data and limited generalizability across cities due to differences in urban structure and movement patterns. *These differences call for rethinking existing modeling paradigms and developing new approaches specifically designed for the geospatial domain.*

In this paper, we outline a vision for GEO-centric foundation models. We decompose the end-to-end modeling pipeline and analyze unique challenges for which existing language-based methods fall short. We also highlight key considerations, including privacy, transferability, and interpretability, that are essential for building robust, general-purpose representations of GEOs.

## 2 Unique Modeling Challenges

### 2.1 Mobility-to-GEO Attribution

The first step in learning mobility-enhanced representations of GEOs includes converting raw GPS traces into sequences of geospatial objects, a process we **call GEO attribution**. This step identifies which GEOs (e.g., POIs, roads, or neighborhoods) are visited, passed by, or generally associated with a given trip. Conceptually, GEO attribution loosely parallels tokenization in NLP, where raw input is segmented into discrete tokens. However, unlike language, where tokens are well-defined and drawn from a fixed vocabulary, geospatial “tokens” must be inferred from continuous spatial traces.

Mapping GPS points to GEOs is an ambiguous, dynamic, and task-specific process. GPS data is often noisy or imprecise, making it difficult to determine which GEOs are truly relevant to a trip. Yet, even with clean trajectories, attribution poses several modeling challenges. For example: *Should we include only explicitly visited GEOs, or also those merely passed nearby?* This decision has important implications. Including too many nearby but irrelevant GEOs could lead to over-attribution, while failing to capture brief but meaningful stops could result in under-attribution (e.g., missing a transit hub due to short dwell time).

Even the attribution methods themselves vary widely. For instance, map matching align GPS points to road networks [34] while POI attribution attempts to associate visits based on spatial and temporal cues. For the latter, existing methods rely mostly on heuristics such as fixed dwell-time thresholds, nearest-neighbor assignments, and spatial buffers, which constrain accuracy [35, 36, 40]. The challenges increase when GEOs serve multifunctional roles (e.g., a transit hub that is also a shopping center), or when semantic importance outweighs proximity; for example, GPS points recorded near a university may be closest to coffee shops and adjacent amenities, yet a longer dwell time suggests the visit should be attributed to the campus [37]. Lastly, unlike deterministic tokenization in language, GEO attribution is context-dependent, as the same trajectory may yield different GEOs depending on whether the goal is routing or behavior analysis. All these challenges make GEO attribution an inherently uncertain and ill-defined problem, that needs to be solved to enable effective GEO representation learning.

### 2.2 GEO Encoding

Once GEOs have been identified, the next step is to convert them into fixed-length vectors suitable for downstream learning tasks. This step, formally referred to as encoding, is loosely analogous to word embedding in NLP, where models like Word2Vec [30] capture semantic similarity based on co-occurrence in text. However, GEO encoding is significantly more complex due to the multimodal nature of geospatial data. GEOs are defined not only by their location in space, but also by their functional roles (e.g., serving as a school or a hospital) and by temporal patterns of interaction, such as when and how frequently they are visited. These diverse attributes must be jointly captured to produce representations that are both meaningful and generalizable. The remainder of this section discusses the challenges and representative methods for encoding GEOs along these three key dimensions: *spatial, contextual, and temporal*.

#### 2.2.1 Spatial Dimension.

Spatial characteristics are central to the identity of GEOs and must be explicitly preserved in their representations. However, encoding spatial data presents unique challenges. First, encoders must support **heterogeneous geometries**, including points, polylines, and polygons. Most existing approaches have focused on point geometries [25], with comparatively limited attention given to polylines and polygons [27, 47]. Although geospatial objects can be converted into alternative formats like images or text to fit standard machine learning pipelines [5, 7, 8, 18, 45], such transformations often discard critical spatial information, such as the object’s exact position in space, which can degrade performance on downstream tasks. Recent efforts like Poly2Vec [39] represent early progress toward a unified encoding framework that preserves spatial characteristics across diverse geometry types.

Second, geospatial data spans **multiple spatial scales**, from neighborhoods to cities and regions, requiring representations that remain robust across varying resolutions. To that extent, some methods operate on a fixed grid scale based on task assumptions (e.g., zip code level prediction) [1, 48], while others adopt hierarchical schemes to encode information across multiple levels [8, 19]. These approaches remain sensitive to grid design and often fail to generalize across scales. Multi-scale encoders offer more flexibility, but current designs are limited to point geometries [26].

Third, spatial encoders should capture a **rich set of spatial properties**. While most existing methods primarily focus on distance-based proximity [16, 18], they should also capture topological (e.g., adjacency, containment) and directional relationships as well as structural characteristics, such as the curvature of a road or the footprint complexity of a region. These properties are essential for enabling geospatial reasoning tasks that go beyond proximity, such as identifying whether a building lies within a hazard zone, or determining whether two roads are connected. Despite their importance, such properties are rarely captured or evaluated in existing pipelines [39]. Addressing these gaps calls for encoders that unify geometry types, support continuous spatial input, and capture rich relational and structural properties.

#### 2.2.2 Contextual Dimension.

GEOs are often associated with rich contextual information that provides semantic grounding beyond geometry alone. This includes **object level** functional roles and categories, such as determining if a GEO is a hospital, a residential building, or a main road, as well as geometry-specific metadata, like the number of floors of a building or traffic volume. These features, often sourced from OpenStreetMap, government records, or remote sensing, are essential for understanding a GEO’s role and should be embedded directly into its representation [5, 11, 19, 23].

Context also extends to the **neighborhood level**, where features such as the distribution of nearby POI types capture a GEO’s functional role within its broader environment. Some studies aggregate neighborhood features using fixed-radius buffers [19, 42], spatial attention mechanisms [8, 26] or graph-based approaches [13, 44, 49]. These signals are crucial for capturing urban structure, functional zoning, and patterns of human activity.

Despite their importance, semantic attributes are often treated as standalone metadata, appended to GEO’s representation without modeling their interaction with the object’s geometry. This limits models ability to capture how meaning arises from the interplay between spatial features and semantics. A key challenge is to design representations that reflect this interdependence. While a few studies explore joint learning between geometry and semantics [5], approaches that explicitly model these relationships remain limited.

#### 2.2.3 Temporal Dimension.

In LLMs, sequential dependencies are captured using position encodings that assume uniformly spaced, discrete tokens [41]. A similar strategy can be applied to GEOs by ordering them based on the time they were visited, providing an initial temporal context. However, visit order alone is insufficient to capture the rich temporal semantics of GEOs, particularly because the meaning of GEOs can change over time. For instance, a single location might function as a coffee shop in the morning and transition into a bar at night, reflecting distinct roles at different times of day. This suggests that GEO representations should be **dynamic**, adapting to temporal context inferred from mobility data.

Designing such temporally adaptive representations remains an open challenge. While most prior work focuses on modeling trajectories as temporal sequences [14, 20, 22], relatively little attention has been paid to how the semantics of individual GEOs evolve over time. This raises a fundamental questions: *Should a single GEO have multiple representations that vary across time?* And if so, *what should the temporal granularity of these representations be?* Temporal behaviors in human mobility are often multi-scale, making it unclear how fine-grained these representations should be and how to aggregate them effectively. Answering these questions is critical for building models that treat time as an integral part of GEO representation, rather than as an auxiliary input.

### 2.3 The Vocabulary Challenge

With GEOs identified and encoded, the subsequent challenge is to determine their representation for effective learning. In LLMs, each word or subword token is assigned a discrete ID from a fixed vocabulary, typically around 128k tokens in size [3, 29]. While the specific tokenization technique matters, the decision principles are clear. One might think that we can similarly assign a unique identifier to each GEO, and learn a corresponding embedding. However, the space of possible objects on the map, is orders of magnitude larger, with hundreds of millions of locations worldwide, and follows an extremely long-tailed distribution. This is further complicated by the fact that some GEOs are frequently visited (e.g., airports, road segments), while others are rarely or never re-visited (e.g., private homes), leading to severe data sparsity for unique identifiers.

Representing each GEO with a dedicated embedding is both computationally prohibitive and will yield poor generalization in data-sparse regions with few visitation patterns. More fundamentally, fixed vocabularies contrast with the continuous nature of geographic space, where new or rarely visited locations are constantly encountered. Some recent methods attempt to bypass discrete identifiers by embedding raw spatial and temporal signals directly [20, 51], or by learning higher-level clusters to represent fine-grained spatial locations within graph neural networks, thereby improving scalability [43]. These initial attempts, though useful, are limited to specific GEOs and downstream tasks, thereby not fully capturing the complexity of geospatial semantics. This raises a fundamental question for mobility-based GEO modeling: *How can we construct representations for a vast, sparse, and continuously evolving set of GEOs without relying on predefined vocabularies?*

### 2.4 Hard(er) Constraints

Unlike language, where any token can, in principle, appear in any position, modeling GEOs through mobility poses fundamental real-world constraints that must be respected to generate realistic representations. We discuss some of these these constraints below.

#### 2.4.1 Accessibility and Reachability.

Not all GEOs in a mobility sequence are equally accessible or reachable; a GPS trace cannot be arbitrarily associated with any GEO [21]. Physical access restrictions (e.g., private buildings, gated facilities), transportation constraints, and temporal feasibility (e.g., whether a location can be reached within a given time window) all affect which GEOs are plausible candidates. For example, a university campus may require an access pass, or a remote trailhead may be inaccessible without a vehicle. These constraints create a non-uniform feasibility landscape over geographic space, requiring models to reason not only about the locations a trajectory has visited, but also about which locations were *realistically reachable* given physical and temporal, and transportation mode constraints.

#### 2.4.2 Capacity and Spatiotemporal Density Limits.

Every GEO has intrinsic limits on how many agents can physically occupy or interact with it over space and time. A concert venue, for instance, cannot accommodate unlimited attendees regardless of demand. Similarly, a multi-story office tower can support far more occupants than a small neighborhood park, even if both have similar ground-level footprints. These capacity constraints are not merely operational considerations; they are fundamental semantic properties that influence how a GEO functions. Failing to account for these constraints can lead models to inaccurately assume that a GEO can support more activity than is physically or operationally feasible, resulting in unrealistic outputs in tasks such as demand forecasting, crowd simulation, or mobility prediction. Accurate GEO representations must therefore account for spatiotemporal density limits to support meaningful and physically plausible inference.

## 3 Potential Impact

We envision GEO representations serving as a fundamental layer for geospatial foundation models (GEOFMs), enabling a wide range of applications across domains, which we group into three categories:

1. **Object-centric tasks** involve reasoning about individual GEOs and their attributes. Examples include improving maps quality [9], like detecting missing or mislabeled POIs, inferring building functions from mobility patterns, identifying access points to large venues (e.g., stadium entrances), and correcting road connectivity errors (e.g., missing links, wrong one-way assignments). Another example is decision support, which includes recommending optimal locations for new businesses based on visitation patterns, estimating the capacity of facilities (e.g., determining parking space capacity), and assisting drivers with context-aware navigation, such as detecting likely entrances or drop-off points near a destination.
2. **Mobility-centric tasks** involve understanding and optimizing movement patterns across space and time. Applications include dynamic traffic management based on real-time mobility data [38], optimizing delivery and service routes, forecasting logistics demand, analyzing commuter flows for transit planning, and identifying mobility bottlenecks or under-served areas in transportation networks.
3. **Population-level tasks** involve aggregating GEO representations across users, time, and space to uncover macro-scale patterns. Applications include assessing mobility equity, identifying tourist activity patterns, estimating demand for public services such as healthcare or transit, monitoring urban growth and land use change [44, 49], detecting disruptions during large events or disasters [4, 50], and informing long-term decisions for infrastructure investment and public service allocation. By learning structured, multi-scale representations of space, time, and function, GeoFMs could unify these capabilities within a single, general-purpose framework.

## 4 Orthogonal Considerations

### 4.1 Privacy

Privacy concerns are critical for GEO representation learning, given the reliance on human mobility data. Individual trajectories, even when anonymized, can often be re-identified through spatio-temporal modeling, posing risks of unintended disclosure. This is especially concerning when handling sensitive locations like private homes, hospitals, and places of worship. Unlike NLP tokens, which are abstract and generally unrelated to individuals, GEOs are grounded in real-world entities and often reflect personal routines. These risks raise an important design question: *Should certain classes of GEOs, such as private residences, be represented at all?* While including them may improve coverage, it not only introduces serious privacy vulnerabilities but also offers limited value for general-purpose tasks. Responsible representation learning may therefore require filtering, abstracting, or omitting sensitive GEOs altogether, alongside the use of privacy-preserving techniques such as differential privacy [2, 17] or federated learning to enable decentralized model training without sharing raw trajectory data across devices [28]. Balancing representational utility with ethical safeguards is essential for deploying trustworthy GeoFMs in practice.

### 4.2 Cross Region Transferability

Publicly available mobility data are typically restricted to specific geographic areas and narrow time spans, making it difficult to obtain comprehensive, large-scale coverage for training. As a result, GEO representations learned from such data risk being overly specialized to the regions and time periods they were derived from.

Cross-region transferability is a core requirement for general-purpose GeoFMs. But transferring representations across regions is challenging due to substantial variation in mobility behavior, land use, and spatial semantics [10, 23, 50]. For example, the distribution of POIs, transportation modes, and urban density in Tokyo differs significantly from that in Los Angeles. Therefore, models trained on region-specific patterns may fail to generalize to areas with different structural or behavioral dynamics.

To support such transferability, GEO representations must go beyond encoding region-specific mobility patterns. They should also encode spatial priors that capture differences in urban form (e.g., density, land use, connectivity), scale (e.g., city vs. neighborhood), and mobility modality (e.g., walking vs. driving), and that can adapt to distributional shifts both in the physical layout of geographic space and in the mobility behaviors associated with it.

### 4.3 Interpretability

As GEO representations grow in dimensionality, they get harder to understand. Yet interpretability remains essential, especially in high-stakes domains such as urban planning, transportation, and public policy, where stakeholders must be able to understand and justify spatial decisions or model-driven recommendations. In language modeling, word embeddings have been shown to align with interpretable semantic axes, such as gender, tense, or country-capital relationships. Similarly, GEO embeddings should aim to reveal dimensions that correspond to meaningful spatio-temporal and contextual attributes [12], but this level of interpretability remains largely underexplored in current research.

Improving interpretability in GEO representations may benefit from techniques adapted from language models. For example, embedding probes can be repurposed to test whether GEO embeddings capture meaningful attributes such as accessibility or population density. Overall, this is a promising direction for building more transparent and accountable GeoFMs, particularly in applications where explainable decision-making is critical.

## 5 Conclusion

In this paper, we introduced GEOs as a unifying abstraction for representing geospatial objects and outlined the core challenges in learning their representations across spatial, contextual, and temporal dimensions. We highlighted the unique technical difficulties of modeling GEOs using human mobility data, including issues like sparsity, scale, transferability, privacy and interpretability. We also explained why GEOs require fundamentally different modeling assumptions than language tokens. We argue that GEOs represent a critical building block for general-purpose GeoFMs, and achieving this goal requires collaborative efforts from the community.

## Figures and Tables

**Figure 1:**
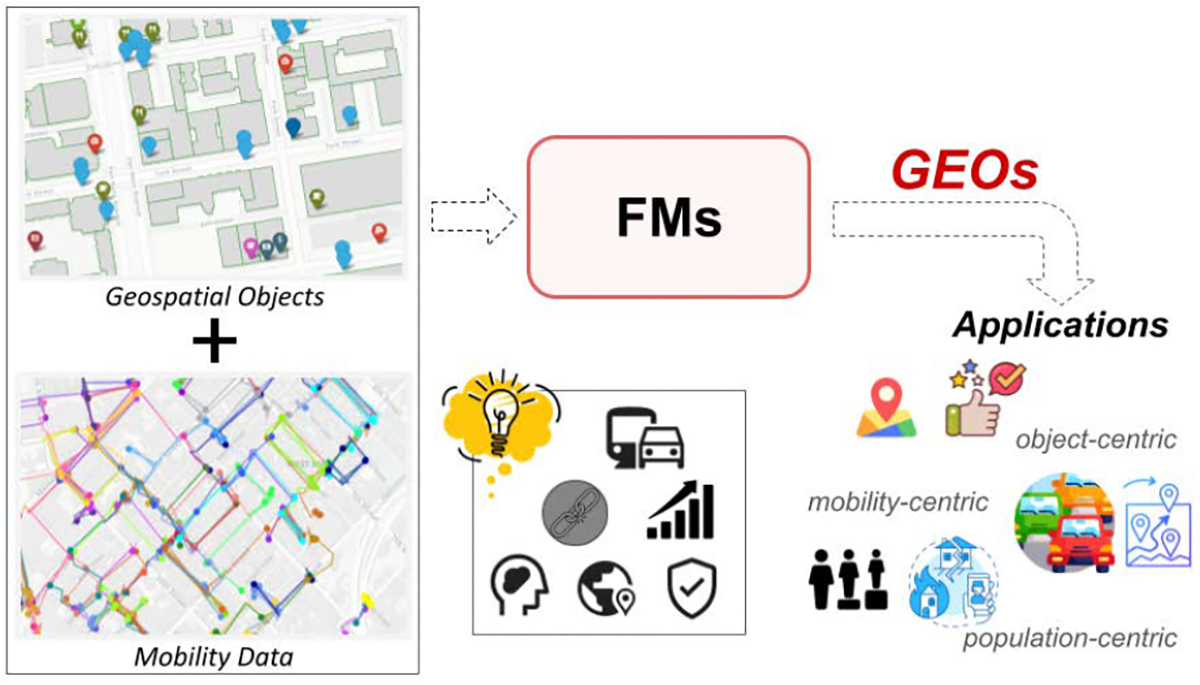
Pipeline overview for mobility-enriched GEOs: approach, applications, and modeling considerations.

## CCS Concepts

• Computing methodologies → Neural networks; • Information systems → Geographic information systems.
